# Bacterial Inoculants Mitigating Water Scarcity in Tomato: The Importance of Long-Term *in vivo* Experiments

**DOI:** 10.3389/fmicb.2021.675552

**Published:** 2021-06-15

**Authors:** Valentina Riva, Francesca Mapelli, Giovanna Dragonetti, Mustafa Elfahl, Lorenzo Vergani, Paola Crepaldi, Nicola La Maddalena, Sara Borin

**Affiliations:** ^1^Department of Food, Environmental, and Nutritional Sciences, Università degli Studi di Milano, Milan, Italy; ^2^Department of Land and Water Division, Mediterranean Agronomic Institute of Bari, IAMB, Bari, Italy; ^3^Department of Agricultural and Environmental Sciences – Production, Landscape, Agroenergy, Università degli Studi di Milano, Milan, Italy

**Keywords:** crop production, drought stress, biofertilizers, plant microbiome, sustainable agriculture, tomato, greenhouse experiment

## Abstract

Global population growth and climate change raise a challenge to agriculture, which, combined with the issues concerning the use of chemical fertilizers, have generated increasing attention in the use of plant-associated bacteria as a sustainable strategy in agri-food systems. The objective of this study is to evaluate the ability of five bacterial strains, previously isolated from the rhizosphere or endosphere of plants adapted to harsh environmental conditions, to act as potential plant biofertilizers in different conditions of water availability. The strain biosafety for a deliberate environmental release was investigated through a literature survey and antibiotic resistance testing. The selected strains were first characterized for their plant growth–promoting (PGP) and rhizocompetence-related traits through *in vitro* assays and then on short-term *in vivo* experiments on tomato plants. A long-term greenhouse experiment was further conducted to monitor the PGP effect of the bacteria during the entire life cycle of tomato plants subjected to full irrigation or to severe water deficit conditions, aiming to assess their actual effect on plant productivity, which is the ultimate target of the agricultural sector. Some of the strains showed a potential in improving water use efficiency and mitigating plant water stress. Under severe irrigation deficit, four of the tested strains, *Micrococcus yunnanensis* M1, *Bacillus simplex* RP-26, *Pseudomonas stutzeri* SR7-77, and *Paenarthrobacter nitroguajacolicus* 2–50, significantly increased the number of productive plants in comparison to non-bacterized control ones. Two of them, *Bacillus simplex* RP-26 and *Paenarthrobacter nitroguajacolicus* 2–50, demonstrated also, under full irrigation, to significantly improve the water productivity in comparison with non-bacterized plants. Despite all the strains showed promising PGP potential in short-term assays, the positive effect of the bacterial inoculants on plant physiology and fruit yield was observed in some cases but never corroborated by statistical significance. These results highlight the importance of performing long-term *in vivo* experiments to define the real PGP ability of a bacterial inoculant to positively impact plant production.

## Introduction

A new revolution in the agricultural sector is needed to sustain and increase the productivity of cultural lands based on fewer intensive inputs having reduced environmental impact (GAP Report, 2018). The intensive agriculture practices, used in the last decades to meet the needs of a rapidly growing population, have high environmental impacts, such as the increase of agrochemical pollution and ecosystem deterioration (Backer et al., 2018; Saleem et al., 2019). Soil microorganisms have been suggested as the backbone of a sustainable food production system with high yield and low input (Saleem et al., 2019). In particular, the exploitation of plant growth–promoting (PGP) microorganisms as biofertilizers has been largely proposed according to the scientific research outcomes and is even supported by leading agrochemical industries (Abhilash et al., 2016; Singh et al., 2016; Timmusk et al., 2017). PGP microorganisms are indeed soil- and root-dwellers that fulfill important functions for plant growth and health by both direct and indirect mechanisms (Berg, 2009; Turner et al., 2013; Compant et al., 2019). PGP microorganism importance on the fertilizer market is constantly increasing, and the compound annual growth rate surged at 12.9% between 2017 and 2025 according to the Transparency Market Research report (2017).

In adverse environmental conditions, in particular, plants have evolved with a complex microbial community enriched in PGP microorganisms able to counteract abiotic stress and to confer a certain level of stress tolerance to the plant host (Soussi et al., 2016; Riva et al., 2019b; Sangiorgio et al., 2020). Plants adapted to extreme environmental conditions, thus, represent an interesting source of PGP microorganisms, which could potentially boost the growth of other plant species, including agricultural crops, improving their ability to cope with abiotic stress (Yuan et al., 2016; Chen et al., 2017). This potential was shown by Soldan et al. (2019) working on mangrove endophytic bacterial isolates able to promote barley and rice plantlet growth under saline conditions. This is of particular interest considering the emerging conditions of limited water resource availability becoming worse by the ongoing climate changes (FAO, 2018).

Plant growth–promoting bacteria have the potential to impart drought tolerance to the plants by several mechanisms including (i) coregulation of the plant hormone balance through the production of indole-3-acetic acid (IAA), involved in the development of the root system, and the degradation of 1-aminocyclopropane-1-carboxylate (ACC), a precursor of the stress hormone ethylene; (ii) production of polysaccharides, which improve the soil water-holding capacity and protect roots from mechanical stress caused by dry soil compactness; (iii) induction of the accumulation of osmolytes and antioxidants; and (iv) transcriptional modulation of different plant stress responsive genes (Vurukonda et al., 2016).

The development pipeline of a novel biofertilizer commonly starts with the screening of large microbial strain collections for various and possibly multiple PGP-related traits evaluated in laboratory-scale tests, often based on *in vitro* assays. In a bottom-up approach, the most promising strains are subsequently tested *in vivo* under greenhouse conditions to select those able to promote plant growth. However, *in vivo* evaluations are often short-term, and the PGP effects are monitored only in the first stages of plant development (Backer et al., 2018; Compant et al., 2019). A critical point often overlooked by research studies is that promising results in terms of PGP do not necessarily turn into increased plant productivity, which is the primary aim in developing PGP inocula for application in agriculture.

In the present work, we evaluated the long-term effects of tomato plant inoculation with potential PGP bacterial strains isolated from the rhizosphere and endosphere of plants adapted to harsh environments. Tomato plants were cultivated in a greenhouse under an optimal water regime and severe water stress condition, and the PGP effect was monitored during the plant cycle up to the obtainment of fruits.

## Materials and Methods

### Phylogenetic and Functional Characterization of the Bacterial Strains Used in the Study

#### Strain Isolation and Identification

Five bacterial isolates were selected for the present study from bacteria collections previously established in our laboratory (Supplementary Table 1). Three strains were isolated from extremophilic plants: (i) *Micrococcus yunnanensis* M1 was isolated from the endosphere of *Avicennia marina* mangrove propagules as described by Soldan et al. (2019). (ii) The endophytic strain *Bacillus simplex* RP-26 was isolated from the leaves of a plant species that survives extreme dehydration, *Selaginella lepidophylla* (unpublished data). (iii) *Pseudomonas stutzeri* SR7-77 was isolated from the rhizosphere of *Salicornia strobilacea*, a halophilic plant collected in a coastal area in Tunisia (Marasco et al., 2016). Two strains, *Paenarthrobacter nitroguajacolicus* 2–50 and *Paenarthrobacter aurescens* 2-T30 (previously belonging to *Arthrobacter* genus, Busse, 2016), were isolated from the rhizosphere of *Centaurea nigrescens* collected in a highly and historically polluted site located in Northern Italy and mainly contaminated with polychlorinated biphenyls and metals (Vergani et al., 2017). The genomic DNA of the isolates was extracted through boiling cell lysis (Ferjani et al., 2015), and the strains were identified through 16S rRNA gene amplification, using universal primers 27F and 1492R, which span nearly the full length of the gene (∼1,400 bp) and sequencing (Macrogen, South Korea). Nucleotide sequences were subjected to BLAST search using the BLASTN program on the NCBI database (accession numbers are indicated in Supplementary Table 1; identification information is indicated in Supplementary Table 2).

#### Biosafety Characterization of the Isolates

The antibiotic resistance phenotype of the strains was characterized by performing a disk-diffusion test in Mueller–Hinton medium (0.7% agar) with six different antibiotics: cephalothin (30 μg), chloramphenicol (30 μg), ciprofloxacin (5 μg), rifampicin (5 μg), tetracycline (30 μg), and vancomycin (30 μg) provided by Laboratorios Conda S.A., Madrid, Spain. After 24 h of incubation at 30°C, the strains were classified as sensitive or resistant according to the interpretative standards provided by Laboratorios Conda S.A. using *E. coli* ATCC 25922 as a reference strain. An extensive literature review was performed on the five species to which the isolates belong in order to exclude safety concerns related to their possible application in the field. The review was based on a literature database web search (Scopus, April 2021) using the same keywords used by EFSA Panel on Biological Hazards (Biohaz) et al. (2017) to include a taxonomic group in the list of biological agents having “qualified presumption of safety” (QPS) status. The research was performed using the specie name crossed with the keyword “toxin^∗^,” “disease^∗^,” “infection^∗^,” “clinical^∗^,” “virulen^∗^,” and “antimicrobial resistan^∗^.”

#### *In vitro* Screening of PGP and Root-Colonization Related Traits

*In vitro* characterization of PGP-related traits was performed on the five bacterial strains. Inorganic phosphate solubilization and ACC deaminase activity as well as the production of ammonia, siderophore, and auxin were assessed as previously described (Penrose and Glick, 2003; Cherif et al., 2015). Isolates were also tested for abiotic stress tolerance, namely their ability to grow in the presence of 20% polyethylene glycol (PEG) (osmotic stress) and at 42°C (heat stress) (Mapelli et al., 2013). Their ability to grow in the presence of different sodium chloride concentrations was investigated using a microdilution method. In a 96-well microtiter, 100 μl of the bacterial suspension and 100 μl of sodium chloride (NaCl, Sigma) dissolved in tryptic soy broth (TSB, Sigma) were added obtaining, respectively, a final concentration of 10^6^ cell/ml and 1, 2, 4, 6, and 8% NaCl. A negative control, containing TSB medium with each of the NaCl dilutions without bacteria and a positive control with inoculated TSB medium without NaCl, were also performed. Each thesis and the correspondent controls were performed in triplicate. The plates were incubated at 30°C for 48 h, and the test was considered positive if turbidity, significantly higher than the negative controls, appeared in the well at the end of incubation time.

Finally, the biofilm production capacity of the isolates, as a proxy for root colonization, was evaluated by a colorimetric assay based on crystal violet staining (Riva et al., 2019a), and the results were evaluated according to the method of Stepanović et al. (2007).

## *In vivo* Assessment of Tomato Growth Promotion During a Short-Term Greenhouse Experiment

Strains *P. aurescens* 2-T30 and *P. nitroguajacolicus* 2–50 were previously demonstrated to promote tomato plant growth during a short-term (30 days) greenhouse experiment (Vergani et al., 2017). The ability of *M. yunnanensis* M1, *B. simplex* RP-26, and *P. stutzeri* SR7-77 strains to promote tomato plant growth were verified in a 60-day experiment on tomato plants artificially subjected to water stress under greenhouse conditions. Tomato seeds (var. *Kamonium F1*-Syngenta^®^) were sown in soil substrate (SER V10-14P VigorPlant^®^, Supplementary Table 3) in trays, and 10 days after germination, uniform sized seedlings were selected, each transplanted in separated 0.2 kg soil substrate pots. The three bacterial strains were inoculated separately on tomato plants (*n* = 5) 3 days after transplantation. The bacterial inocula were prepared by centrifuging at 4,000 rpm for 15 min the cell cultures grown for 24 h at 30°C in TSB medium and suspending the pellet in sterilized physiological solution (0.9% NaCl) with a final bacterial concentration of 10^8^ cell/gram of soil. Non-inoculated plants (*n* = 5) were irrigated with the same amount (10 ml) of sterile distilled water and considered as negative control. Water stress was induced by interrupting the irrigation for 20 days, starting from 9 days after bacterial inoculation, and it was repeated a second time, after a break of 8 days in which plants were regularly irrigated. Sixty days after sowing, plants were harvested for the measurements of shoot/root length and fresh/dry weight.

### Long Term Plant Growth Promotion Assays Under Full and Deficit Irrigation

#### Experimental Design and Setup

The five selected strains were tested for their *in vivo* PGP ability on potted tomato plants under full irrigation and severe water deficit conditions. A long-term experiment was performed under semi-field conditions (no controlled humidity and temperature parameters, Supplementary Figure 1) in a greenhouse located at the Mediterranean Agronomic Institute of Bari (IAMB) for the entire life cycle of tomato plants (7 months, from January to July 2017). Tomato plants (*n* = 120 in total) were subjected to a full irrigation regime (*n* = 60) and a severe induced water deficit corresponding to 50% of the plants’ water requirement (*n* = 60). Pots were irrigated every 2 days to restore water losses induced by the actual evapotranspiration (AE) demand. AE was obtained considering the difference between the water soil content measured before the irrigation and the pot water capacity, which was assumed as the optimal water availability and defined for the different tomato stages (vegetative, flowering, and fruiting) through the time domain reflectometry technique (Topp et al., 1980). An oscilloscope (Tektronix 1502C) was used to acquire the soil water contents data through a time domain reflectometry probe installed in three pots per each water irrigation regime. To assess the accuracy of this technique, the same pots were weighted with an electronic balance. The full irrigation regime was set to the amount of water lost by AE while water stress was induced offsetting 50% of the AE.

#### Plant Growth and Bacterial Inoculation

Tomato seeds (var. *Kamonium F1*-Syngenta^®^) were germinated in polystyrene plug trays filled with soil substrate. After 40 days, the most uniform sized seedlings were selected and planted in pots, each filled with 9.5 kg of soil collected from a IAMB experimental field and 0.5 kg of pumice placed on the bottom to facilitate water drainage. Soil chemical and physical characteristics were analyzed. In detail, the analyses showed a silty loam texture, including 14.8, 20.5, and 64.7% of sand, clay, and loam, respectively (USDA, soil taxonomy), an organic matter content equal to 1.21% and pH 8.4.

Tomato plants were subjected to two bacterial inoculations with the same bacterial strains during the vegetative stage and in the middle of the growing season, 7 and 83 days after transplantation (dat), respectively. Bacterial inocula were prepared as follows: cells were grown in TSB liquid medium for 24 h at 30°C and bacteria cell concentration was estimated using a Thoma cell counting chamber. The bacteria culture was then centrifuged at 4,000 rpm for 15 min, and the pellet was suspended in sterilized physiological solution to obtain a final bacterial concentration of 10^7^ cell/g of soil. Fifty milliliters of each bacterial suspension were applied to the soil surrounding the collar of potted tomato plantlets (*n* = 20 for each bacterium). According to previous tests, we estimated a total amount of 200 g as the soil surrounding the root system in the collar zone to be considered as the soil amount recipient of the bacterial suspension. Negative control non-bacterized plants (*n* = 20) were watered with the same volume of sterile distilled water. One week after the first plant bacterization, the water deficit (50% of AE) was applied on half of the replica plants for each bacterium and the negative control (*n* = 10). The same bacterization procedure was applied at mature fruiting stage at 83 dat.

#### Assessment of Plant Growth Promotion and Yield Measurement

Four measurement campaigns (at 26 dat = vegetative stage, 46 dat = flowering stage, 81 dat = fruit setting stage, and 108 dat = mature fruiting stage, according to Jones, 2007) were performed in correspondence with different plant growth stages to monitor the physiological parameters of the plants on five replicas for bacterized plants and for negative control at the two different irrigation regimes. Leaf photosynthesis, stomatal conductance, and transpiration rate were measured by the LI-COR 6400 portable photosynthesis system. The index of leaf water use efficiency in the photosynthesis process (WUE) was evaluated as the photosynthesis/transpiration ratio. At the end of the experiment, root and shoot length and fresh and dry root weight were measured to determine the plant growth. Five different fruit harvest campaigns (92, 99, 106, 118, 130 dat) were performed collecting the mature fruits from all 120 plants. The optimal harvest time was identified based on two indices, absorbance difference (IAD) and color, determined with two non-destructive methods: visible and near infrared spectroscopy (DA meter) and light absorption (chroma meter), respectively. Chemical characteristics of tomato juice as pH and Brix degree were measured on a subset of fruits with the aim to assess the accuracy of the two non-destructive methods: the pH was measured using a pH meter, and Brix degree was measured with a hand refractometer. Diameter and weight of the collected fruit were recorded and the tomato yield per plant, average fruit weight per plant and number of fruits per plant was calculated. Moreover, to define tomato yield per unit of water consumption, the water productivity (WP) index was calculated as the ratio of yield/evapotranspiration (Molden et al., 2010).

#### Statistical Analyses

Statistical analyses were carried out using JMP13^®^ software (SAS Institute, Cary, NC, United States). Two-way ANOVA was performed to evaluate the interaction between the two factors “water regime” and “bacteria” and the effect of the “water regime” factor on data. One-way ANOVA and *post hoc* Dunnett’s analyses were performed to assess the effects on plant growth and productivity of the bacterial inoculation in comparison with the negative control treatment under the two different water regimes. An exact binomial test was performed for qualitative analyses considering the number of plant able/not able to produce tomatoes under water stress condition.

## Results

### Strain Selection and Biosafety Evaluation

The bacterial strains used in the present study belonged to five different species and were selected among collections of bacterial isolates obtained from plants naturally adapted to cope with different harsh environmental conditions (i.e., salinity, desiccation, chemical contamination).

In light of a potential release of these bacteria in the environment as biofertilizers, some evidence of their biosafety were obtained. An extended literature search on the five species did not find any document reporting their involvement in pathogenicity toward humans, animals, or plants. The antibiotic resistance profile of the strains was analyzed on six antibiotics selected to investigate different mechanisms of action. The strains, overall, demonstrated scarce antibiotic resistance. *M. yunnanensis* M1 and *B. simplex* RP-26 were susceptible to all the tested antibiotics, and *P. aurescens* 2-T30 and *P. nitroguajacolicus* 2–50 showed resistance to ciprofloxacin (5 μg), and *P. stutzeri* SR7-77 was resistant to vancomycin (30 μg) and cephalothin (30 μg) (Table 1).

**TABLE 1:** *In vitro* screening of the isolates used as inocula in pot greenhouse experiment.

**Isolate ID**	**PGP traits**					**Abiotic stress tolerance**							**Biofilm**	**Antibiotic resistance**					
	**ACC-d**	**Auxin**	**P Sol.**	**Sid.**	**NH*3***	**42°C**	**20% PEG**	**1% NaCl**	**2% NaCl**	**4% NaCl**	**6% NaCl**	**8% NaCl**	**OD 610 nm**	**RA**	**VA**	**CIP**	**KF**	**TE**	**C**
*Micrococcus yunnanensis* M1	+	+	−	−	+	−	+	+	+	+	+	−	1.803	s	s	s	s	s	s
*Bacillus simplex* RP-26	+	+	+	-	+	+	+	+	+	+	+	−	0.301	s	s	s	s	s	s
*Pseudomonas stutzeri* SR7-77	−	+	−	+	+	+	+	+	+	+	+	−	0.398	s	R	S	R	s	s
*Paenarthrobacter aurescens* 2_T30	−	+	−	−	−	+	+	+	+	−	−	−	0.340	s	S	R	s	s	s
*Paenarthrobacter nitroguajacolicus* 2_50	+	−	−	−	−	−	+	+	+	−	−	−	0.350	s	S	R	s	s	s

**In vitro* PGP tests: ACC-d = ACC deaminase activity; Auxin = auxin production; P Sol. = phosphate solubilization; Sid. = siderophore production; NH3 = ammonia production. Abiotic stress tolerance: 42°C = heat stress; 20% PEG = osmotic stress. Tolerance to NaCl: 1%, 2%, 4%, 6%, 8%. Biofilm = colorimetric assay for biofilm formation: strains are considered positive for biofilm formation if OD_610_ > 202 (cutoff value calculated based on negative control OD). Antibiotic resistance to RA = rifampicin; VA = vancomycin; CIP = ciprofloxacin; KF = cephalothin; TET = tetracycline; C = chloramphenicol: R = resistant, s = sensitive.*

### PGP and Root Colonization–Related Traits of the Selected Bacterial Strains

The bacterial traits involved in root development, stress perception, and nutrient uptake improvement were screened with *in vitro* test. In particular, the isolates were tested for the ability to produce auxin, a phytohormone that promotes root system growth (Backer et al., 2018), and for the ACC-deaminase (ACC-d) activity that reduces the negative effect of ethylene, the plant stress-related hormone, by degrading its precursor (Nascimento et al., 2018). *M. yunnanensis* M1 and *B. simplex* RP-26 were able to produce auxin and displayed ACC-d activity; *P. stutzeri* SR7-77 and *P. aurescens* 2-T30 were able to produce auxin while *P. nitroguajacolicus* 2–50 showed ACC-d activity (Table 1). Regarding the nutrient uptake improvement, *M. yunnanensis* M1, *B. simplex* RP-26, and *P. stutzeri* SR7-77 produced ammonia, the latter being also able to produce siderophores, and *B. simplex* RP-26 was able to solubilize phosphate (Table 1). The strains were further tested for additional traits that could help them to survive under environmental conditions typical of arid soils: all strains were able to grow in the presence of 20% PEG; thus, they should be able to counteract the osmotic stress, and *B. simplex* RP-26, *P. stutzeri* SR7-77, and *P. aurescens* 2-T30 were also able to grow at 42°C, which indicates their tolerance to temperature fluctuations (Table 1). Moreover, *M. yunnanensis* M1, *B. simplex* RP-26, and *P. stutzeri* SR7-77 were able to grow in the presence of relatively high salt concentrations up to 6% NaCl, and *P. aurescens* 2-T30 and *P. nitroguajacolicus* 2–50 tolerated at maximum 2% NaCl (Table 1). Finally, the bacterial isolates were subjected to a colorimetric assay to verify their potential biofilm production capability, a trait relevant for root surface colonization. Considering the cutoff value of 0.202 (calculated on negative control OD_610_), all the strains showed potential biofilm production capability. In particular, *M. yunnanensis* M1 was a strong biofilm producer (OD_610_ = 1.803, more than four times the cutoff value), and the other strains can be considered, according to the method of Stepanović et al. (2007), weak biofilm producers because their OD_610_ ranged from the cutoff value to two times the cutoff value (Table 1).

During a short-term, 30-day greenhouse experiment, the strains *P. aurescens* 2-T30 and *P. nitroguajacolicus* 2–50 were demonstrated to significantly increase shoot length (up to 38 and 37%, respectively) and fresh weight (up to 73 and 68%, respectively), and *P. nitroguajacolicus* 2–50 also promoted the root length up to 47% (Vergani et al., 2017; Supplementary Figure 2, panel A and B). *M. yunnanensis* M1, *B. simplex* RP-26, and *P. stutzeri* SR7-77 strains were shown to significantly promote the growth of tomato plants suffering water stress during the short-term greenhouse experiment performed in this study (*p*-value < 0.05, Supplementary Figure 2, panel C and D). In particular, *M. yunnanensis* M1 improved shoot length and fresh weight up to 16 and 38%, respectively; *B. simplex* RP-26 improved root fresh weight and shoot dry weight up to 36 and 42%, respectively; *P. stutzeri* SR7-77 improved shoot dry weight up to 42%.

### Plant Growth Promotion of Tomato Plants in Soil Under Full Irrigation and Water Stress Conditions

In a long-term greenhouse experiment performed under semi-field conditions, tomato plants were cultivated for the entire life cycle, up to fruit production, under full irrigation or severe water stress conditions (given by 50% irrigation deficit). All the parameters, at different experimental times, were subjected to the saturated two-way ANOVA model. Because the interaction between the two factors, “water regime” and “bacteria” was not significant (*p*-value > 0.05, Supplementary Table 4) for all the measured parameters and the water regime factor showed as strong effect an as we could expect (Supplementary Table 4), the interaction was removed from the model to assess the effects of the bacterial inoculation on plant growth and productivity.

**FIGURE 1:**
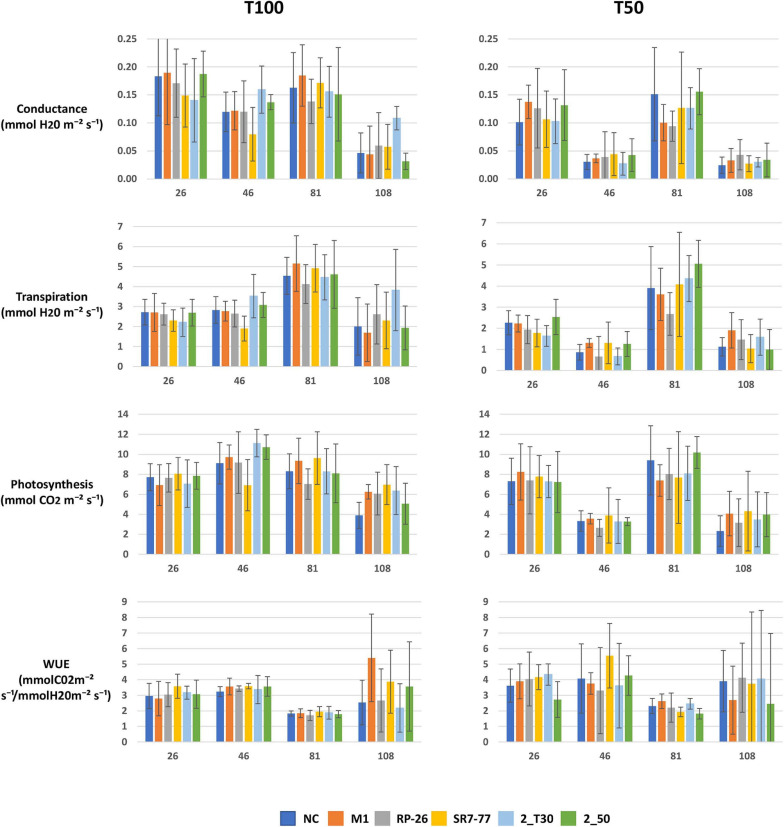
Effect of PGP bacteria inoculation on tomato plant physiological parameters (i.e., stomatal conductance, transpiration rate, photosynthesis, and leaf WUE) of plants grown under full irrigation condition (T100) and water stress (T50) during different plant growth stages: 26 = 26 dat, vegetative stage; 46 = 46 dat, flowering stage; 81 = 81 dat, fruit setting stage; 108 = 108 dat, mature fruiting stage. Data are represented as average value (*n* = 5) with bars referred to standard deviation. NC, non-bacterized control plants; PGP bacteria strains: *Micrococcus yunnanensis* M1, *Bacillus simplex* RP-26, *Pseudomonas stutzeri* SR7-77, *Paenarthrobacter aurescens* 2_T30 and *Paenarthrobacter nitroguajacolicus* 2_50.

The effect on tomato plant growth of (i) the artificially induced water stress and (ii) the bacterial strain application was evaluated first by measuring physiological parameters (i.e., net leaf photosynthesis, transpiration rate, and stomatal conductance) at times corresponding to vegetative, flowering, fruit setting, and at the mature fruiting stages. As expected, plant–gas exchange (represented by stomatal conductance and transpiration rate) was significantly reduced (*p*-value < 0.05, Supplementary Table 4) under water stress along all the life cycle of tomato plants, and the photosynthesis rate was significantly reduced under water stress at 46 and 108 dat (flowering and mature fruiting stages, respectively). Bacterial inoculations had no significant effect on plant physiological parameters, neither under full irrigation nor under water stress (Figure 1 and Supplementary Table 5). The leaf WUE, calculated as the ratio between photosynthesis and transpiration, increased under the water stress condition, in particular, it significantly increased at 26 and 81 dat (vegetative and fruit setting stages, respectively) (Supplementary Table 4). Leaf WUE was not significantly influenced by bacterial inoculation under both water regime conditions (Figure 1 and Supplementary Table 5).

After the fruiting stage, at the end of the tomato plant growing season, root and shoot length and fresh and dry root weight were measured (Table 2). Plant biomass was strongly influenced by water availability (*p*-value < 0.0001, Supplementary Table 4): in control plants (NC) under the water stress condition, root and shoot length were reduced by 28 and 35%, respectively, and fresh and dry root weight were reduced by 53 and 55%, respectively. Concerning the bacterial strains, none of them had significant effects on plant biomass comparing the same water regime (Supplementary Table 5).

**TABLE 2:** Effect of PGP bacteria inoculation on plant growth under full irrigation condition (T100) and water stress (T50).

		**root length (cm)**	**shoot length (cm)**	**fresh root weight (g)**	**dry root weight (g)**
**T100**	**NC**	16.2 ± 1.79	100.2 ± 17.57	8.352 ± 3.08	2.988 ± 0.85
	**M1**	17.8 ± 2.02	98.2 ± 12.71	10.188 ± 2.10	3.224 ± 0.59
	**RP-26**	17.5 ± 3.00	97.9 ± 18.90	8.436 ± 3.99	2.66 ± 0.48
	**SR7-77**	17.1 ± 1.14	87.7 ± 7.05	8.366 ± 3.19	2.828 ± 0.33
	**2_T30**	18.4 ± 3.97	95 ± 9.47	10.156 ± 4.88	3.188 ± 0.53
	**2_50**	17.7 ± 2.54	90.3 ± 9.30	8.782 ± 2.85	2.99 ± 0.26
**T50**	**NC**	11.7 ± 1.15	65.3 ± 6.50	3.948 ± 1.44	1.356 ± 0.31
	**M1**	10.9 ± 1.52	61.8 ± 12.87	3.800 ± 1.62	1.232 ± 0.42
	**RP-26**	11.9 ± 2.19	66.4 ± 7.11	4.004 ± 1.31	1.334 ± 0.37
	**SR7-77**	11 ± 1.77	65.1 ± 9.77	3.61 ± 1.69	1.272 ± 0.44
	**2_T30**	10.7 ± 2.41	64 ± 11.57	3.586 ± 1.63	1.294 ± 0.45
	**2_50**	12.6 ± 2.48	62.8 ± 8.84	3.554 ± 1.33	1.52 ± 0.38

*Data are indicated as average value (*n* = 5) ± standard deviation. NC: non-bacterized control plants; strains: *Micrococcus yunnanensis* M1, *Bacillus simplex* RP-26, *Pseudomonas stutzeri* SR7-77, *Paenarthrobacter aurescens* 2_T30 and *Paenarthrobacter nitroguajacolicus* 2_50.*

**FIGURE 2:**
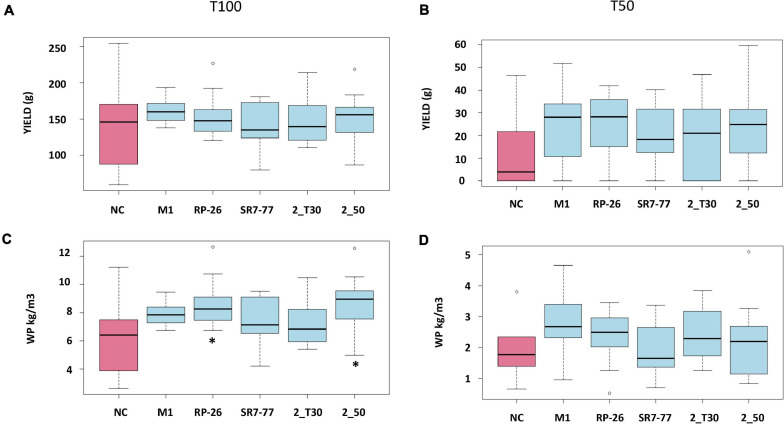
Effect of PGP bacteria inoculation on **(A,B)** yield and **(C,D)** WP of tomato plants (*n* = 10) under full irrigation condition **(A,C)** and water stress **(B,D)**. Red box indicates non-bacterized control plants (NC); blue boxes indicate plants bacterized with *Micrococcus yunnanensis* M1, *Bacillus simplex* RP-26, *Pseudomonas stutzeri* SR7-77, *Paenarthrobacter aurescens* 2_T30, *Paenarthrobacter nitroguajacolicus* 2_50. Data on the left panels refer to full irrigation (T100), and panels on the right refer to water stress condition (T50). Stars indicate statistical significance according to ANOVA test.

**FIGURE 3:**
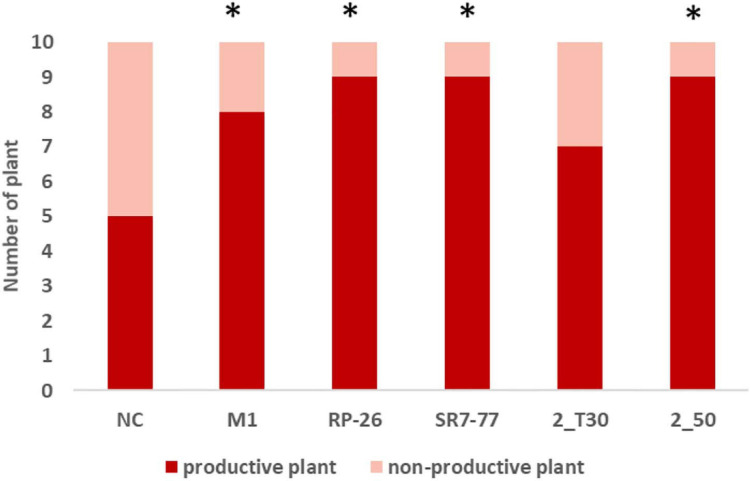
Number of productive and non-productive tomato plants under the water stress condition. Red bars indicate the number of plants that produced at least one tomato; pink bars indicate the number of plants that did not produce any tomatoes. Stars indicate statistical differences in comparison with NC (*p*-value < 0.05; exact binomial test). NC, non-bacterized control plants; *Micrococcus yunnanensis* M1, *Bacillus simplex* RP-26, *Pseudomonas stutzeri* SR7-77, *Paenarthrobacter aurescens* 2_T30, and *Paenarthrobacter nitroguajacolicus* 2_50 strains.

Tomatoes were harvested during five campaigns (92, 99, 106, 118, and 130 dat) once mature. Maturity of the harvested fruits was confirmed by measuring Brix degrees and pH on a subset of tomatoes. The measured pH was 4 ± 0.09, and Brix degrees were always higher than 5, which is the commodity-related value for commercialized tomatoes (Barrett et al., 2007). Water stress strongly influenced plant productivity (*p*-value < 0.0001, Supplementary Table 4). Almost all bacterized tomato plants showed higher average yield (*n* = 10) in comparison with control NC plants (Figure 2, panels A and B) with an evident effect, in particular, under the water deficit condition. Tomato yield, expressed as grams of fruit per plant, increased by 51% in plants bacterized with *M. yunnanensis* M1 and *B. simplex* RP-26, 50% in plants bacterized with *P. nitroguajacolicus* 2–50, and 42 and 41% in plants inoculated with *P. aurescens* 2-T30 and *P. stutzeri* SR7-77, respectively. Also, the average fruit weight per plant and number of fruits per plant of bacterized plants were almost double in comparison to the NC plants under the water deficit condition (Supplementary Figure 3, panels C and D). The observed differences were, however, not supported by statistical significance (Supplementary Table 5), likely due to the reduced number of fruits available for the analyses. The applied water stress was indeed very strong, inducing the production of a maximum amount of four tomatoes per plant against a maximum of nine tomatoes harvested from plants cultivated under the optimal irrigation regime. In particular, only 50% of control (NC) plants were able to produce at least one tomato under the water stress condition (Figure 3). Comparing the number of bacterized and control productive plants grown under water stress, it was possible to observe a positive and significant effect of the bacterization with four out of five strains, namely *M. yunnanensis* M1, *B. simplex* RP-26, *P. stutzeri* SR7-77, and *P. nitroguajacolicus* 2–50 (Figure 3 and Supplementary Table 6). Moreover, it is interesting to observe that inoculated tomato plants produced mature fruits earlier than non-bacterized control plants (Supplementary Figure 4): earlier fruit production can, thus, represent an advantage, especially when water availability is low.

Under full irrigation, bacterization did not show a statistically significant increase of fruit productivity, when compared with control plants: yield (Figure 2, panel A), number of fruits per plant and average fruit weight per plant, were not significantly influenced by the bacterial inoculum (Supplementary Figure 3, panels A and B). The only parameter significantly improved by the presence of the bacterial inoculum was the WP, which represents the ratio between yield and water consumption (Figure 2, panel C). Particularly, plants inoculated with *B. simplex* RP-26 and *P. nitroguajacolicus* 2–50 improved WP up to 29 and 30%, respectively (*p*-value = 0.0159 and *p-*value = 0.0129, respectively) (Table 3). Under the water stress condition, all the bacterized plants, with the exception of those inoculated with *P. stutzeri* SR7-77, showed an average WP higher than control plants although not significantly (Figure 2, panel D).

**TABLE 3:** Comparison of the effect of PGP bacteria inoculation on WP of tomato plants under full irrigation condition.

**(A)**					
	**Df**	**Sum Sq**	**Mean Sq**	***F* value**	**Pr(> F)**

THESIS	5	49.31267	9.86253	2.7973	**0.0256**
Residuals	54	190.38657	3.52568		
**(B)**					
**DUNNETT’S TEST**					

**Groups**		**Abs(dif)-LSD**		***p*-value**	

Paenarthrobacter nitroguajacolicus 2_50		0.44		**0.0129**	
Bacillus simplex RP-26		0.375		**0.0159**	
Micrococcus yunnanensis M1		−0.44		0.1598	
Pseudomonas stutzeri SR7-77		−0.81		0.3567	
Paenarthrobacter aurescens 2_T30		−1.19		0.6572	
NC (non-bacterized control)		−2.18		1.0000	

*Significant differences between bacterized plants and non-bacterized control plants are indicated in bold. **(A)** main test: ANOVA; **(B)***post hoc* test: Dunnett’s test.*

## Discussion

The five selected bacterial strains, isolated from salt-, desiccation-, and contamination-tolerant plants (Marasco et al., 2016; Vergani et al., 2017; Soldan et al., 2019), showed tolerance to abiotic stresses such as osmotic and heat stress, determined as the ability to grow on PEG and at high temperatures, indicating their adaptation to thrive in harsh environments. In addition, *M. yunnanensis* M1, *B. simplex* RP-26 and *P. stutzeri* SR7-77 were able to grow in the presence of high salt concentration (6% NaCl), an interesting trait because salt stress is often related to drought. The capability of the abovementioned strains, compared with *P. aurescens* 2-T30 and *P. nitroguajacolicus* 2–50, to grow at higher salt concentrations can be explained by their original environment of isolation, i.e., endosphere/rhizosphere of plants growing in saline and arid conditions. The PGP potential of the selected isolates was initially analyzed in this study through *in vitro* tests. The two *Paenarthrobacter* strains showed potential biostimulant capacity, producing auxin or degrading ACC: under drought stress conditions, these two activities can improve plant growth by promoting root system development and expansion for the absorption of the greatest amount of water and by reducing the plant stress perception (Riva et al., 2019b). Besides biostimulant abilities, *M. yunnanensis* M1, *B. simplex* RP-26, and *P. stutzeri* SR7-77 also showed the potential to improve the nutrient uptake through ammonia and siderophore production or phosphate solubilization. The *in vitro* screening of bacterial strains for PGP-related traits is the first step of a bottom-up approach in the development of new biofertilizers. However, to recognize the best PGP candidates, the *in vivo* experiments are fundamental because bacteria that demonstrate weak PGP potential *in vitro* were, in some cases, the best performers *in vivo* (Cardinale et al., 2015).

Besides PGP potential, we also characterized the strains in terms of biosafety, considering that microbial biofertilizers are intended to be deliberately released in the environment, to exclude the strains showing potential biosafety risks from further plant testing. For this purpose, we included the antibiotic resistance profiles in the characterization of the five selected inoculant strains. The potential risks related to the occurrence of antibiotic resistance genes (ARGs) in the genome of PGP bacterial strains is widely overlooked in most of the research works (Ramakrishna et al., 2019). However, there is growing evidence that the overuse of antibiotics in animal husbandry and the manure application to agricultural soils introduce antibiotic residues and cause the spread of antibiotic resistant bacteria (ARB) and ARGs in the environment and soil (Riber et al., 2014; Blau et al., 2019). Moreover, the rhizosphere is regarded as a hot spot of microbial activity and gene transfer: hence, ARB can transfer ARGs, often located on mobile genetic elements, to other bacteria inhabiting soil and plant, threatening animal and human health (Blau et al., 2019). We believe that it is crucial to avoid large-scale deliberate introduction of beneficial bacteria into soil, which could exacerbate the spread of ARGs in the environment (Kang et al., 2017). We tested the resistance of the five bacterial strains to six antibiotics that span different mechanisms of action, finding out that antibiotic resistance is poorly detectable in these strains. However, our study does not provide information on the resistance mechanisms and the possible genetic marker and mobile genetic elements associated with the observed phenotype. The real risk evaluation in the case of their massive environmental release as biofertilizers would, therefore, need further studies. The extended literature search about the five species, carried out following EFSA guidelines for the QPS on biological agents (Herman et al., 2019), did not point out biosafety issues. Moreover, the five strains investigated in this study are phylogenetically affiliated to bacterial species that, according to a reference document provided by the German Committee on Biological Agents^1^ that classified prokaryotes into risk groups, belong to risk group 1, and for this reason their use in field conditions does not imply particular concern on safety.

In the aftermath of these considerations, the PGP potential of the five bacteria was tested *in vivo* on tomato plants, a species of pivotal agricultural interest and particularly sensitive to water stress (Dodds et al., 1997; Le et al., 2018). Tomato was cultivated in optimal conditions, under full irrigation, and under severe water deficit to evaluate the effect of bacterial inoculants in drought mitigation. The effect of irrigation deficit on tomato plants was first analyzed because it is known that the plant itself may cope with water scarcity through different physiological strategies (Chaves et al., 2003; Egea et al., 2018), which explain, for instance, the increase of leaf water use efficiency under water stress observed in the current study. The data collected concerning physiological parameters, plant growth and production of non-bacterized control plants overall confirmed that *Kamonium* tomato plants were suffering due to water deficit conditions with the exception of photosynthesis rate data at 26 and 81 dat (vegetative and fruit setting stages, respectively) that were not significantly different between the two irrigation conditions. This can be considered in agreement with the literature, provided that the photosynthetic capacity of plant experiencing drought shows no or little change (Cornic and Massacci, 1996).

Differently from our expectation based on *in vitro* and short-term experimental results, the effects of bacterial inocula on plant physiological and growth parameters were not statistically significant under both full irrigation and water deficit conditions for none of the strains. The variability observed within the same experimental thesis (water regime + inocula) was high, and this could have influenced the statistical analysis even when the presence of bacteria seemed to improve plant performance according to the trends of measured parameters. This highlights the need to design experiments with a number of replicates higher than the five specimens used in this work for each tested treatment/condition. All five bacterial strains demonstrated the ability to significantly increase biomass in short-term experiments on 30-day tomato seedlings (Vergani et al., 2017) or on 60-day tomato seedlings suffering water stress although in our work they did not significantly improve shoot and root length and weight of tomato plants at the end of the life cycle. These results underline the importance of performing long-term PGP *in vivo* experiments (Compant et al., 2019). *In vivo* evaluations of PGP effects are often short-term, monitoring the plant growth and physiology parameters only in the first plant life stages. Nevertheless, the length of the monitoring is a determinant in the evaluation of the PGP effect because a promotion effect exerted only in the first weeks of the plant cycle does not necessarily correspond to a better performance in the late life stages and especially in crop production, which is the ultimate goal of biofertilizer administration.

A recent work highlights the importance of applying PGP bacteria at specific stages of plant development when the plant truly requires it (He et al., 2019). The physiological parameters data collected on tomato plants during this experiment revealed that plants particularly suffered water stress during the flowering stage (e.g., conductance, transpiration, and photosynthesis data at 46 dat in Figure 1). We can, thus, hypothesize that administration of PGP inoculants could have been more helpful for plant growth if applied at that development stage.

The PGP effect of selected bacterial species on tomato yield of plants experiencing drought has been positively demonstrated in other research works. It is reported by Le et al. (2018), which used, in an open field experiment, a commercial bacterial biofertilizer (a mixture of *P. putida*, *A. chroococcum*, *B. circulans*, and *B. megaterium* named Phylazonit) and by Van Oosten et al. (2018), which showed the ability of *Azotobacter chroococcum* 76A to improve the yield of MicroTom tomato plants under salt stress conditions during a greenhouse experiment. In the present work, all the bacterized plants showed a higher average tomato yield in comparison to the non-bacterized ones under both full irrigation and water deficit conditions although these results were not corroborated by statistical analysis. A significant effect of PGP bacteria inoculation was observed on the number of productive plants under the water deficit condition: Although half of the non-bacterized control plants were completely not productive, almost all the inoculated plants produced at least one fruit.

The productivity of tomato plants was found to be generally quite low in comparison with the standard production of the *Kamonium* variety. Not only did plants subject to drought stress have limited fruit production (due to the applied water deficit condition), but also plants grown under optimal irrigation had a lower yield than the standard ones that range between 300 and 500 g/plant. Possibly, the absence of fertilization and the high temperature registered in the greenhouse, especially during the last 3 months of the experiment influenced plant production: Optimal temperature for tomato range, generally, within 21 and 28°C in daytime and 15 and 20°C at night (Filgueira, 2008). However, the non-optimal conditions adopted in this study to grow tomato plants were intentionally established aiming to increase the possibility to detect the effect mediated by bacteria inocula.

In our specific context, the evaluation of the bacteria in terms of PGP effect was realized also looking at the water productivity, a crucial index to evaluate drought resistance in plants (Kang et al., 2009; Gholamhoseini et al., 2013). Under water stress conditions, four out of five bacteria improved WP if compared with non-bacterized control plants. The improvement was statistically significant only under full irrigation conditions for tomato plants inoculated with two out of the five strains (*B. simplex* RP-26 and *P. nitroguajacolicus* 2–50), which induced an increase of fruit yield with lower evapotranspiration rate.

In conclusion, with the present study, we observed that plant-associated bacteria tolerant to abiotic stresses showed promising results in terms of plant productivity, in particular under severe water deficit conditions. In this condition, four out of the five strains statistically increased the number of productive plants. Two of these strains (*B. simplex* RP-26 and *P. nitroguajacolicus* 2–50), moreover, significantly improved water productivity under the full irrigation condition.

However, most of the results based on physiological, growth, and production parameters need to be confirmed by statistical analyses. Future experimental designs should account for more controlled-environment greenhouse conditions and for the increase of biological replicate numbers to overcome the high data variability, which possibly masked the differences we expected to detect between bacterized and non-bacterized control plants. On the other side, field experiments could overcome the low productivity obtained from potted and stressed plants, which lead to the production of a limiting number of fruits. The results of this study underline that PGP activity demonstrated on a given strain based on *in vitro* traits or short-term *in vivo* assays observing only the first plant life stage will not necessarily provide a real benefit in terms of plant products.

## Data Availability Statement

The datasets presented in this study can be found in online repositories. The names of the repository/repositories and accession number(s) can be found in the article/Supplementary Material.

## Author Contributions

SB and NL study was conceived. GD and ME carried out the greenhouse experiment. VR, FM, GD, and LV performed the laboratory work and data analysis. PC and VR performed statistical analyses. VR, FM, and SB interpreted the data and prepared the manuscript. All authors critically reviewed and edited the manuscript and have approved its publication.

## Conflict of Interest

The authors declare that the research was conducted in the absence of any commercial or financial relationships that could be construed as a potential conflict of interest.

## Footnotes

1 www.baua.de/abas
