# Supplementary material for: Bacterial Inoculants Mitigating Water Scarcity in Tomato: The Importance of Long-Term in vivo Experiments
Source: Front Microbiol. 2021 Jun 15;12:675552. doi: 10.3389/fmicb.2021.675552 (PMC8239394; doi:10.3389/fmicb.2021.675552)
Supplement: Supplementary file 1 [file Data_Sheet_1.PDF]

Supplementary Material

Supplementary Table1. Description of the bacteria isolates. Phylogenetic identification (see Supplementary Table 2 for details), accession number of the isolates' 16S rRNA gene sequences in the public nucleotide database ENA, plant species of origin, plant fraction and cultural medium used for isolation (see Materials and Methods section for further information; MM: mineral medium).

Phylogenetic identification	ACC number	Plant species	Plant fraction	Medium
Micrococcus yunnanensis M1	LT838170	*Avicennia marina*	Endosphere	869 1:10
Bacillus simplex RP-26	LT838171	*Selaginella lepidophylla*	Endosphere	R2A
Pseudomonas stutzeri SR7-77	LT838176	*Salicornia strobilacea*	Rhizosphere	King'sB
Paenarthrobacter aurescens 2_T30	LT838178	*Centaurea nigrescens*	Rhizosphere	TSA 1:10
Paenarthrobacter nitroguajacolicus 2_50	LT838179	*Centaurea nigrescens*	Rhizosphere	MM + biphenyl

Supplementary Table2. Identification of the bacteria isolates through 16S rRNA gene sequencing: 16S rRNA identification and accession number of the closest relative species, percentage of identity and length of the nucleotide sequence.

CODE	16S-ID (closest relative species)	ACC n (closest relative)	%	LENGHT
M1	*Micrococcus yunnanensis*	NR_116578	100	1395/1395
RP-26	*Bacillus simplex*	KX301311	99	1423/1424
SR7-77	*Pseudomonas stutzeri*	JN228326	99	1409/1414
2-T30	*Paenarthrobacter aurescens*	JN662517	99	1380/1387
2-50	*Paenarthrobacter nitroguajacolicus*	EF206344	100	1393/1393

Supplementary Table 3. Physical-chemical characteristic of the soil substrate used in the short-term greenhouse experiment

**Physical-chemical characterization of the soil substrait**	
• pH	6
• EC	0.25 dS/m
• Density	110 Kg/mc
• Porosity (v/v)	92%
• NPK 1000g + microelements + chelated Fe	
• Composition: fine Irish peat, blond block peat 0-10 mm, blond block peat 10-20 mm, black peat and clay	

Supplementary Table 4. Two-way ANOVA analyses on the interaction of the two factors “water regime” and “bacteria” and the effect of “water regime” factor on the measured parameters. Significant differences are indicated in bold.

	**“water regime” * “bacteria”**		**“water regime”**	
	F ratio	P value	F ratio	P value
**Plant physiological parameters**				
Conductance 26 dat	0.1635	0.9747	10.7200	**0.002**
Conductance 46 dat	1.7125	0.1498	79.6985	**<0.0001**
Conductance 81 dat	0.7650	0.5795	5.9056	**0.0189**
Conductance 108 dat	1.4919	0.2101	7.6908	**0.0079**
Transpiration 26 dat	0.2714	0.9265	11.2438	**0.0016**
Transpiration 46 dat	2.4366	0.0515	101.9760	**<0.0001**
Transpiration 81 dat	0.4464	0.8138	4.9435	**0.0309**
Transpiration 108 dat	1.1627	0.3412	11.1215	**0.0017**
Photosynthesis 26 dat	0.2554	0.9350	0.0001	0.9913
Photosynthesis 46 dat	2.1927	0.0704	169.7967	**<0.0001**
Photosynthesis 81 dat	1.0277	0.4121	0.0003	0.9858
Photosynthesis 108 dat	0.2695	0.9276	14.2386	**0.0004**
WUE 26 dat	0.1682	0.9731	11.7630	**0.0013**
WUE 46 dat	0.7479	0.5916	2.4163	0.1266
WUE 81 dat	1.4757	0.2153	19.6100	**<0.0001**
WUE 108 dat	0.3833	0.8578	0.0306	0.8619
**Plant growth parameters**				
Root length	0.6559	0.6584	101.6813	**<0.0001**
Shoot length	0.4694	0.7971	104.4254	**<0.0001**
Fresh root	0.3261	0.8948	58.9625	**<0.0001**
Dry root	0.7163	0.6143	180.0935	**<0.0001**
**Production parameters**				
Fruit diameter	0.9130	0.4724	62.8498	**<0.0001**
Fruit weight	0.7335	0.5986	56.5726	**<0.0001**
g of tomato/plant	0.1416	0.9821	582.0054	**<0.0001**
Average fruit weight/plant	1.5595	0.1777	83.1669	**<0.0001**
WP	1.4039	0.2299	295.3040	**<0.0001**

Supplementary Table 5. One-way ANOVA analyses to verify the effect of "bacteria" factor on plant growth and productivity parameters under full irrigation conditions (T100) and water stress (T50). Significant differences are indicated in bold.

	**T100**		**T50**	
	F ratio	P value	F ratio	P value
**Physiological parameters**				
Conductance 26 dat	0.4339	0.8204	0.4867	0.7827
Conductance 46 dat	1.7372	0.1645	0.2735	0.9231
Conductance 81 dat	0.5065	0.7684	0.8775	0.5109
Conductance 108 dat	1.6172	0.1936	0.5045	0.7699
Transpiration 26 dat	0.5364	0.7467	0.6961	0.6315
Transpiration 46 dat	2.5144	0.0515	0.3394	0.8839
Transpiration 81 dat	0.4213	0.8292	0.5052	0.7694
Transpiration 108 dat	1.2946	0.2991	0.6817	0.6417
Photosynthesis 26 dat	0.3401	0.8835	0.1156	0.9877
Photosynthesis 46 dat	2.5135	0.0518	0.3451	0.8803
Photosynthesis 81 dat	0.8397	0.5348	0.6875	0.6376
Photosynthesis 108 dat	1.7726	0.1567	0.3909	0.8501
WUE 26 dat	0.5033	0.7708	0.5398	0.7442
WUE 46 dat	0.3215	0.8951	0.5905	0.7073
WUE 81 dat	0.4337	0.8206	1.0990	0.3864
WUE 108 dat	1.6377	0.1883	0.5584	0.7307
**Plant growth parameters**				
Root length	0.4181	0.8315	0.6760	0.6457
Shoot length	0.6889	0.6366	0.1550	0.9764
Fresh root	0.3271	0.8916	0.0837	0.9941
Dry root	0.7790	0.5746	0.3234	0.8939
**Production parameters**				
Fruit diameter	0.3686	0.8700	0.8043	0.5503
Fruit weight	0.5837	0.7125	0.5927	0.7056
g of tomato/plant	0.4689	0.7977	0.9597	0.4506
Average fruit weight/plant	0.4029	0.8447	1.5122	0.2014
WP	2.7973	**0.0256**	0.6675	0.6503

Supplementary Table 6: Exact binomial test for the analyses of plant productivity under water stress condition. Significant differences between bacterized plants and non-bacterized control plants are indicated in bold with a star

	N° of plants	N° of productive plants	P-value
Micrococcus yunnanensis M1	10	8	**0.044***
Bacillus simplex RP-26	10	9	**0.01***
Pseudomonas stutzeri SR7-77	10	9	**0.01***
Paenarthrobacter aurescens 2_T30	10	7	0.12
Paenarthrobacter nitroguajacolicus 2_50	10	9	**0.01***
NC (non bacterized control)	10	5	-

Supplementary Figure 1. Data of temperature (A) and relative humidity (B) registered during the long-term greenhouse experiment (from March to July 2017)

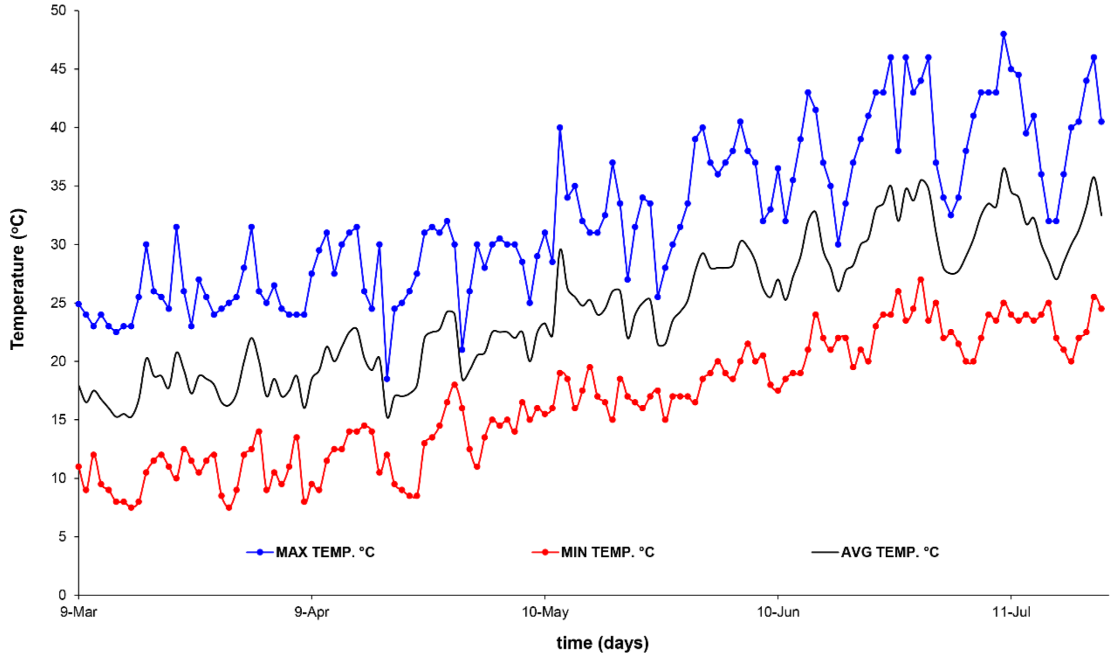

A)

B)

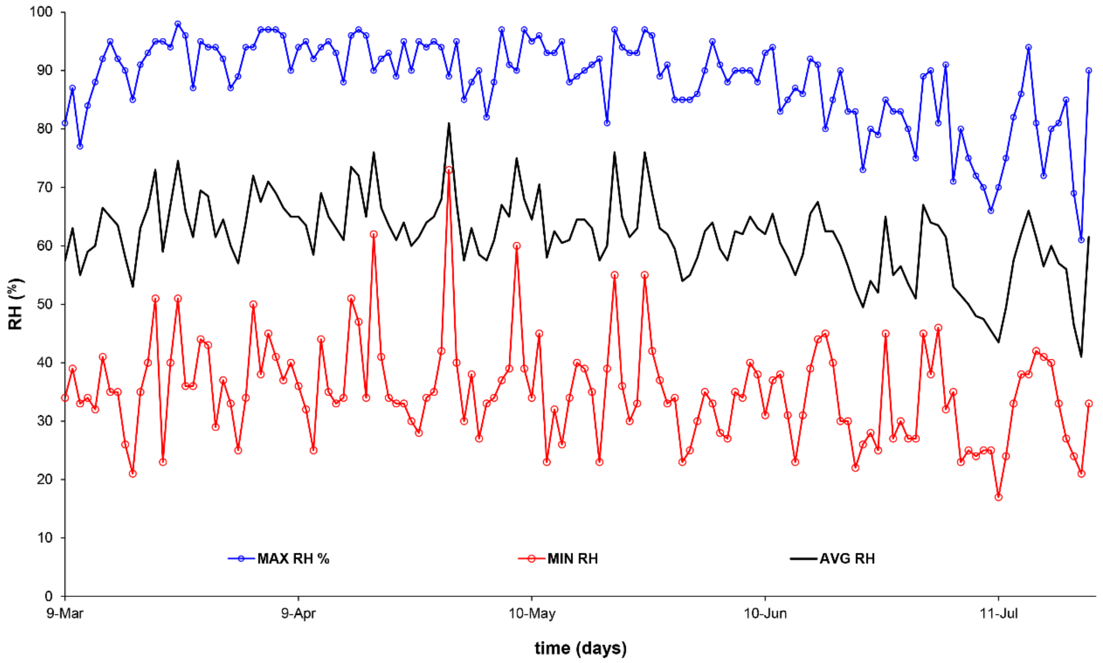

100
90
80
70
60
50
40
30
20
10
0
RH (%)
MAX RH %
MIN RH
AVG RH
9-Mar
9-Apr
10-May
10-Jun
11-Jul
time (days)

Supplementary Figure 2. Results of the two short-term *in vivo* PGP tests on tomato plant. Panel A) and B) show results of *P. aurescens* 2-T30 and *P. nitroguajacolicus* 2-50, while panel C) and D) show results of *M. yunnanensis* M1, *B. simplex* RP-26 and *P. stutzeri* SR7-77. NC = non bacterized control plants. The data were calculated as average of 5 plants per treatment. The star indicates statistically significant differences (p value < 0.05).

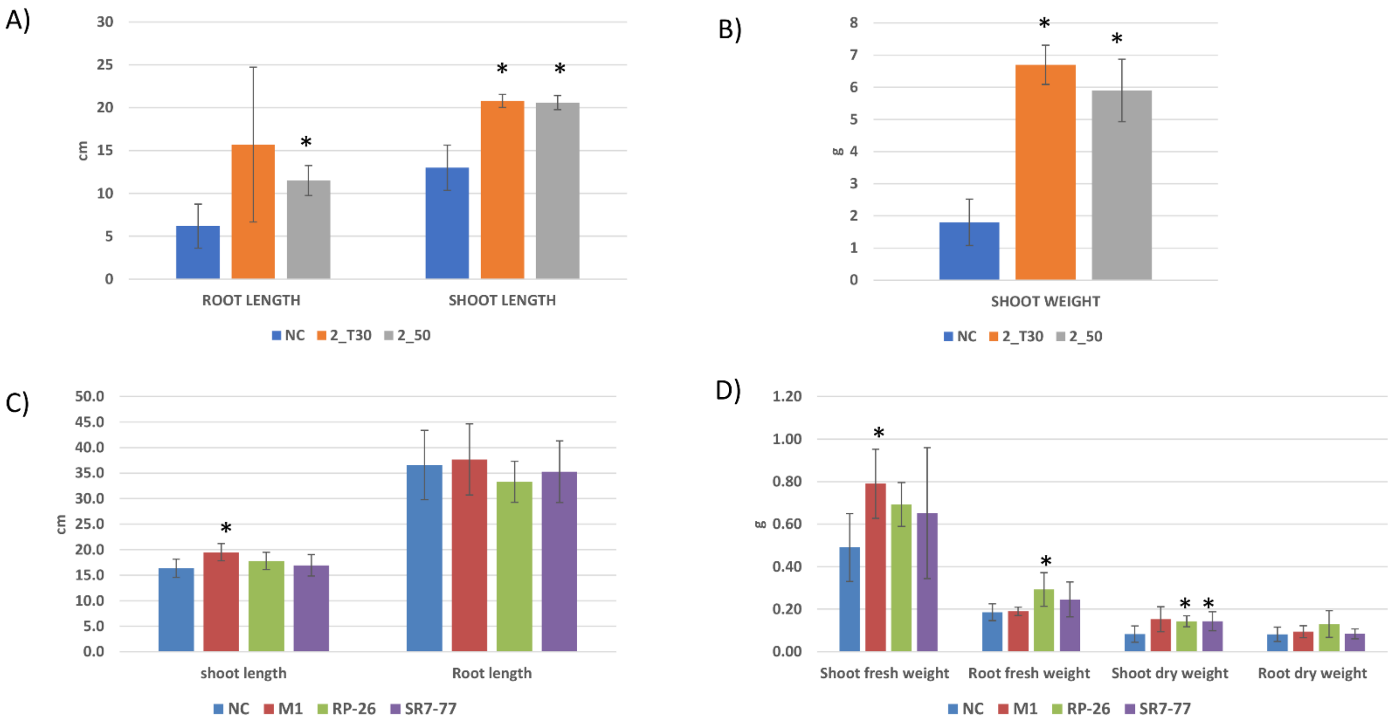

Supplementary Figure 3. Number of tomatoes per plant (A, C) and average tomato weight per plant

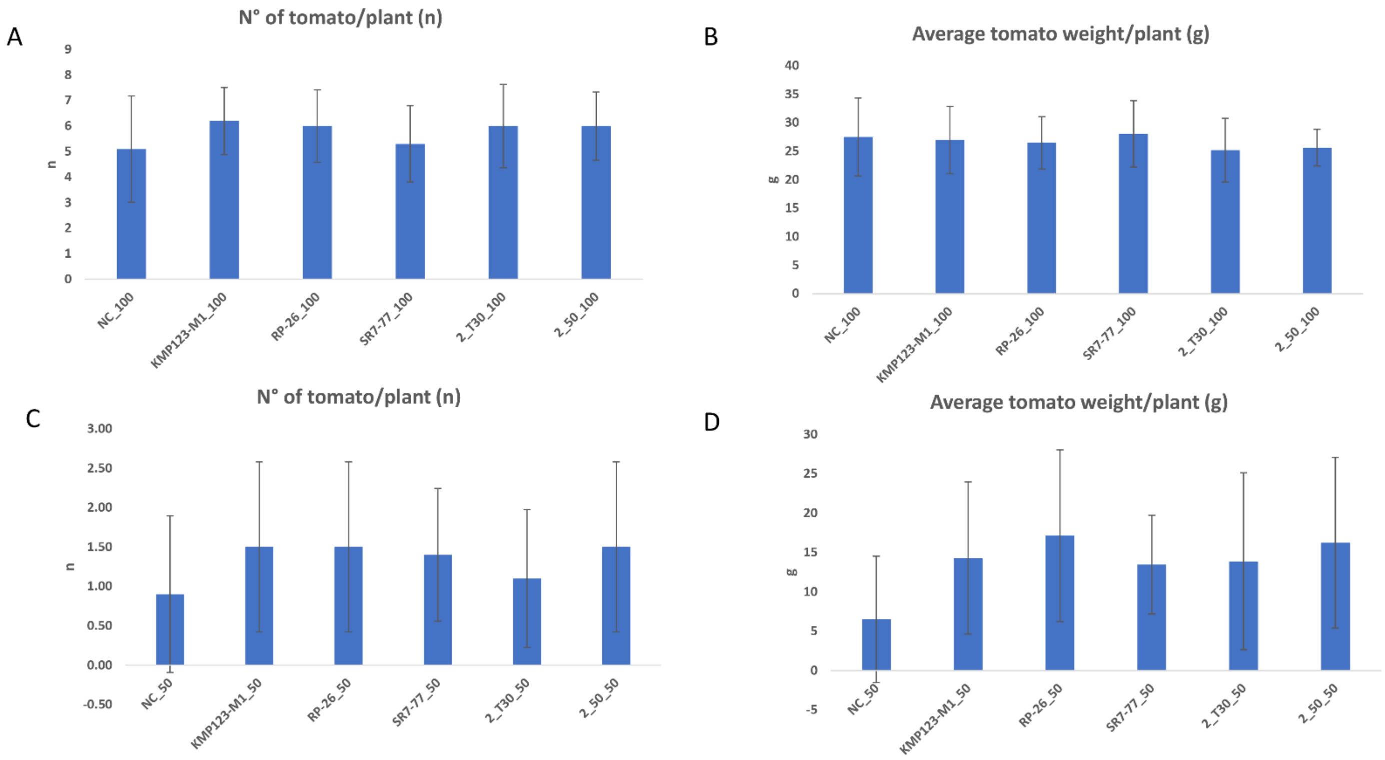

(B, D) under optimal irrigation condition (A, B) and under water stress condition (C, D).

Supplementary Figure 4. Number of fruits collected at five different fruits harvest campaigns: 92 dat, 99 dat, 106 dat, 118 dat and 130 dat, from plants subjected to optimal irrigation condition (T100)

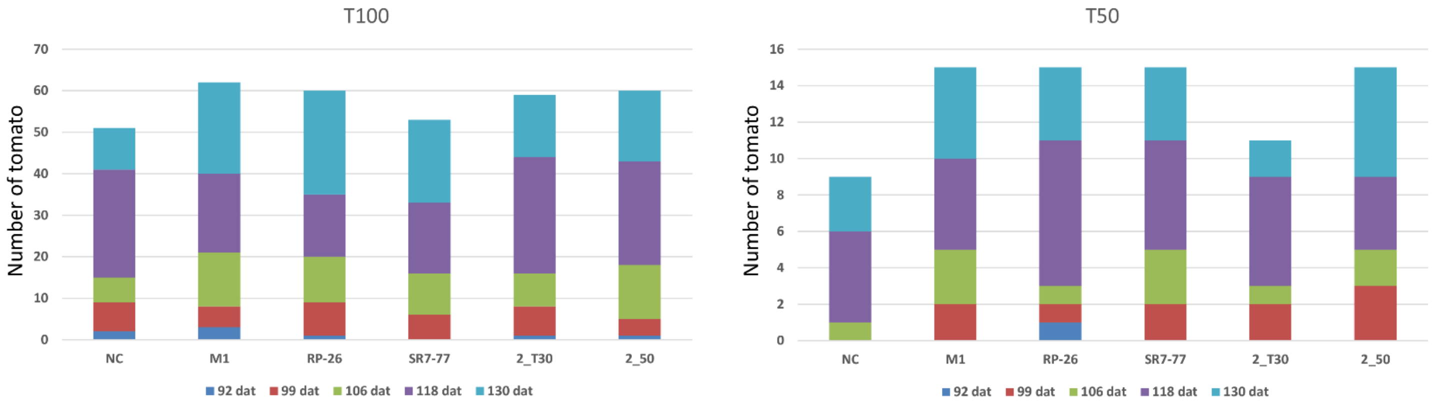

and water stress condition (T50).
